# Herbs as an Active Ingredient in Sport: Availability and Information on the Internet

**DOI:** 10.3390/nu14132764

**Published:** 2022-07-04

**Authors:** Juan F. Garcia, Soledad Arribalzaga, Raquel Díez, Cristina Lopez, M. Nelida Fernandez, Juan J. Garcia, M. Jose Diez, Jesús Seco-Calvo, Matilde Sierra, Ana M. Sahagún

**Affiliations:** 1 Department of Mechanical, Informatics and Aerospatiale Engineering, University of Leon, 24071 Leon, Spain; jfgars@unileon.es; 2 Physiotherapy Department, Institute of Biomedicine (IBIOMED), Campus de Vegazana, University of Leon, 24071 Leon, Spain; marisolarribal@gmail.com; 3 Department of Biomedical Sciences, Institute of Biomedicine (IBIOMED), Veterinary Faculty, University of Leon, 24071 Leon, Spain; rdielz@unileon.es (R.D.); clopcd@unileon.es (C.L.); mnferm@unileon.es (M.N.F.); jjgarv@unileon.es (J.J.G.); mjdiel@unileon.es (M.J.D.); msiev@unileon.es (M.S.); a.sahagun@unileon.es (A.M.S.); 4 Psychology Department, Faculty of Medicine, Visiting Researcher of Basque Country University, 48900 Leioa, Spain

**Keywords:** medicinal herbs, caffeine, turmeric, ginseng, cannabidiol, internet availability, sport supplements

## Abstract

The use of supplements containing herbal active ingredients in sport has increased in recent years. Their consumption is explained by the benefits they may provide and because their natural origin do not involve health complications, from the point of view of the consumers. The aim of this study is to analyze the availability of four supplements (caffeine, turmeric, ginseng, cannabidiol) on the internet and understand the nature of these websites. A descriptive, observational, and cross-sectional study design was used. A detailed search was carried out with specifically developed software. The searches and data evaluation took 10 days. The websites consulted correspond to those that sell supplements, or some sport websites in the case of the Spanish ones, whereas those in English belong to pharmacies, parapharmacies, or herbalists. It is concluded that the websites do not provide adequate information to ensure proper consumption and lack advice on the choices of supplements and their administration guidelines.

## 1. Introduction

The use of herbal supplements has increased in recent years [1,2,3,4]. Among those, *Eurycoma longifolia* Jack [1], *Tribulus terrestris* [5], and others such as ginseng and curcumin have gained interest among athletes for the benefits reported for several years [2].

The herbal products used are mainly derived from leaves, seed extracts, berries, or roots [2]. These products contain phytochemicals that provide the benefits in health and sports practice. Due to their natural components, most of them are considered food and not medicines. Likewise, the pharmacological effects of some of them require a regulation that specifies the protocol of use [6,7].

Herbal supplements are consumed by all layers of populations, but they are most frequently used by athletes, from amateurs to elite level, together with vitamin/mineral supplements as a way of enhancing muscle growth and fat burning [6,7]. Medicinal herbs are used by the active population for multiple reasons: increasing alertness and inducing weight loss or other metabolic responses that enhance performance [6]. Herbold et al. [4] concluded that 17% of female athletes used herbal supplements to increase muscle mass and/or burn fat. The frequency of consumption of these supplements was at least once a month (65.4%). The increase in performance is not the only motivation for their consumption, as improving health is also a strong reason [8].

The prevalence of athletes’ use of supplements varies from 40 to 70%, depending on the study considered [9]. Nevertheless, the growing population of recreational users and amateur athletes has greatly expanded the potential consumers of these supplements. According to the Council for Responsible Nutrition (CRN), the use of herbal supplements has progressively increased in the USA recently, reaching 44% in 2020 [10], probably due to the consumer perception that natural equals healthy. This fact may lead to a false sense of security, as it would be possible to consume banned substances inadvertently in these supplements; additionally, in certain cases, they may become dangerous for health. The easy access to these supplements through the internet, which makes possible for anyone to freely purchase these products all over the world, may also explain their explosive increase in the last decades.

One of the most used supplements is ginseng (*Panax ginseng*, Asian variant). The active ingredients in this medicinal herb are a mixture of triterpene saponin called ginsenosides. The American variant (*Panax quinquefolium* L.) contains higher levels of total ginsenosides, specifically of the ginsenoside subclasses Rb1 and Re, than the Asian one. Ginseng acts on the hypothalamic–pituitary–adrenal cortex axis, minimizing the catabolic effect of cortisol [6]. In sports, it is consumed to increase the intensity of exercise and/or reduce fatigue [11]. Its positive effects are associated with improvements in aerobic capacity, affecting metabolic, cardiovascular and hematological aspects [12].

Equally popular is curcumin, whose main component is diphenoylmethane, with lesser components being demethoxycurcumin and bisdemetoxicurcumin [13,14]. It also consists of 3-5% curcuminoid, responsible for its biological activity [15]. Curcumin is an antioxidant, and due to this property, it has anticancer effects [16], among others. Its antioxidant action is due to the enzymes catalase, superoxide dismutase and glutathione peroxide [17]. Curcumin also activates the glutathione S-transferase complex by inhibiting the generation of free radicals [15], and it possesses anticancer [18], antibacterial [19,20,21], anti-inflammatory [22], anti-aging [23] and neuroprotective activity [24].

On the other hand, cannabidiol (CBD) comes from the *Cannabaceae* family [25]. CBD is a biologically active cannabinoid with anticonvulsant [26], spasmolytic [27] and anticancer [28] effects that is indicated in the treatment of various diseases [29,30,31]. Its biological activity is due to the interaction with the endocannabidoid system, which includes two cannabinoid receptors coupled to G proteins (CB1, located in the central nervous system, and CB2, located in the lymph nodes) and two endogenous (anandamide and 2-arachidonoylglycerol) compounds [32,33,34]. This system participates in the regulation of appetite, pain, mood, inflammation, insulin sensitivity and fat metabolism [34]. CBD acts as an inverse agonist by joining to CB1 and CB2 receptors, and it may be used in the treatment of pain [33]. Regarding this active ingredient, research in the sports field is still limited [31]. Some sports studies have analyzed the effects of this supplement on performance at the end of an aerobic exercise [35,36], although more studies are required to understand its physiological, biochemical and psychological effects in sports [37]. Corroon and Phillips [38] reported that between 27 and 42% of users consumed CBD supplements to improve sleep, but the most frequent use reported is for medicinal treatment (61.56%), followed by improving well-being in general (38.44%).

The latest supplement analyzed in the study is caffeine. This supplement is one of the most studied, and it has shown improvements in performance not only in various sports modalities but also in different aspects of sports performance [39,40,41,42] such as endurance, speed, muscle strength and cognitive function.

At the same time, it is necessary to consider not only the ease of access to these supplements but also that they may be adulterated, even with prohibited agents [2]. Despite the latter, it should be taken into account that research regarding their adverse effects is still needed. Such is the case with CBD, which has been reported to trigger neurotoxicity in the central nervous system, hepatocellular lesions, alterations in the male reproductive system and hypotension in animals [43,44,45], but its effects in humans are unknown. For example, when used in the treatment of epilepsy, CBD develops pharmacological interactions leading to diarrhea, fatigue and drowsiness [46,47]. Regarding ginseng, adverse effects occur in the thyroid and adrenal gland. Ran et al. [48] concluded that the contraindications of this supplement are linked to their dosage: the higher the dose, the worse the effects. On the other hand, this supplement also interacts with monoamine oxidase inhibitors (MAOIs) (antidepressants) and with medications for blood pressure, heart conditions or coagulation disorders [49].

Regarding curcumin, some studies described adverse effects mainly on the liver, due to either toxicity [50,51] or decrease in liver weight [51]. These effects were evaluated in animals with oil extracts or ethanolic extract of turmeric, so that potential complications in humans beyond abdominal pain or flatulence are not known yet, whereas in human studies, capsules were the most frequent dosage forms [52]. Finally, as for caffeine, side effects are linked to high-dose consumption, causing high levels of anxiety and gastrointestinal discomfort [41]. This is partly due to genetic factors, specifically polymorphisms of the adenosine receptor [53]. For this reason, and considering the interindividual variability in the ergogenic response to acute caffeine intake, its recommendation should be individualised [42,54].

On the other hand, it should be considered that research on this topic is still limited. So, it is necessary to corroborate the adverse effects of these supplements, as most of them are sold over the counter. That is, studies are needed on doses and periods of substitution in the most significant samples to be properly known.

Thus, the objective of this study was to evaluate the availability of these supplements in sports through the internet and analyze the characteristics of the websites offering them. Our hypothesis is that athletes have easy access to these supplements and that they are not always consumed properly, which poses a risk not only in sports but also in health.

## 2. Materials and Methods

This study was carried out by a multidisciplinary working group that included pharmacology experts (professors from the University of León, Spain: M.S., J.J.G., M.J.D., A.M.S., R.D. and M.N.F.), an expert in sports medicine (physiotherapy professor from the University of León, Spain: J.S.), an expert in sport nutrition (SA) and a member with expertise in cybersecurity (School of Industrial, Informatics and Aerospatiale Engineering: J.F.G.). J.F.G. developed the search software and programmed the searches. The pharmacology experts reviewed and evaluated in pairs the data obtained. Any potential disagreement was resolved by all members. The searches and the data evaluation took 10 days.

### 2.1. Design

A descriptive, observational and cross-sectional study evaluating the online availability of the four compounds used as ergogenic aids selected in this study, cannabidiol, curcumin, caffeine and ginseng, was conducted. The Strengthening the Reporting of Observational Studies in Epidemiology (STROBE) Statement was used to report data [55] and the checklist included in the material (see Appendix A).

### 2.2. Data Obtained from the Website Analyzed

The following characteristics of the websites found were assessed: number of websites selling the four compounds; number of websites selling each of the products; and type of website, that is, if it was focused on the sale of nutritional supplements; it was a specific website of sport; it had a specific section for sports or nutrition; it belonged to a supermarket, a pharmacy or a parapharmacy; it was a laboratory; or it was a website that sold any type of products. In addition, we checked if they collected the following information: manufacturing laboratory and the country of production of the supplement, recommendations on ergogenic aids or sports supplements, the protocol of administration and their adverse reactions.

For each of the four compounds studied, we evaluated the type of website (considering the categories just mentioned), the number of units offered, and the pharmaceutical forms offered (capsules, compressed/molding tablets, vials, ampoules, sachets with water, solution, bar, drink, drink gel, jelly beans, powder, root, cream, oil, tincture, infusion, chewing gum, seed, oral drops, oral spray, or others, such as patches or liposomes).

### 2.3. Search Engine

We gathered all data from the internet using a modified version of our web crawler, Mirkwood, a parallel focused web tool specifically designed and implemented to make information harvesting much faster, efficient, and secure.

From an architectural point of view, Mirkwood is designed to run in a computer cluster (several machines with a common network for communication), deploying a forest containing spider nests, each of them spawning a discrete number of spiders: The forest is the core of the software (an application written in Java SE running in the main machine of the cluster); the nests (subsets of our software also written in Java) run in the individual machines of the cluster, one nest for each machine (preferably a physical one, but they work with virtual machines too); finally, each spider is implemented as an independent thread within its nest (and thus runs in its same host machine). Each individual spider is ultimately in charge of crawling a given website for information. We use MPI for forest–nests communication and regular thread mechanisms (mainly mutexes and semaphores) for nest–spider communication. Although our software is designed for parallel architectures, it can also work (albeit slowly) in a single machine: in this case, the forest would produce a single nest with *n* spiders in it, and all of them would run in that machine. For further architectural and technical details of our tool, please see [56]. For an example of use of our crawler, you can read our previous study on antibiotics [57].

From a functional point of view, our crawler first identifies the websites to visit in a process called “seed extraction”. To do so, it relies on search engines to find websites containing specific terms (i.e., we look for websites that sell specific plants). After all websites (the “seeds”) have been identified, they are automatically visited to look for the list of the different plants’ names and categories within each of them. Finally, human experts review the crawler’s results to validate them, confirming they really sell a product or substance relevant for our research.

The crawler was improved and updated for this study taking into account our previous experiences to increase the quality of the results obtained. After having investigated the reasons behind this underperformance, we concluded that it was due to Google introducing variations in their search engine’s code, which was becoming increasingly restrained to both location and personalization as of a few years ago. This is clearly an intentional move from their perspective (after all, part of their revenue comes directly from ads, which work better if they are properly tailored to each individual user), but it goes directly against our research intentions: we wanted to find as many relevant websites as possible, not necessarily the ones located (or hosted) near us locally or regionally and not influenced by our (or other users’) previous search queries about the same topic.

By using private browsing, not allowing cookies and forcing the use of Google’s country-independent version, the search engine site had worked well to a point [56,57,58], but it is no longer effective as of 2022. For this research, we had to introduce new technological features, namely VPN usage, multi-country searches and information fusion.

We used VPNs from servers located around the globe (in the US, India, UK, Spain, Germany, Malaysia, Brazil and Colombia, among others). We chose those countries because we considered them important for their geographical proximity or because of the number of internet users (in absolute number) they account for. Note we did not include China, despite having the most online users [59] because of their censorship policies [60].

Then, from every VPN location, we accessed the country-nonspecific Google Search engine version (not really 100% nonspecific anymore, but better than the officially country-specific results for variety). Finally, we combined the results obtained by our crawler in each case into a single results table, eliminating duplicates.

In order to replicate websites’ information extraction from the internet, a computer (preferably a cluster of them) that can run a properly configured web crawler must be used. Even though our software (the crawler), is not free, a working copy of it can be sent by email upon request to the author (J.F.G.) only for research purposes (for-profit or commercial use of the software is strictly forbidden).

## 3. Results

Table 1 shows the characteristics of the websites reviewed. After the search, the software located 237 web addresses in Spanish and 173 in English, of which 27 and 38 were discarded, respectively, for different reasons: (a) They did not connect; (b) they were websites with information about these products, but they did not sell them; (c) they only sold ginseng with the indication of male invigoration; (d) the website had a sports nutrition section (carbohydrates, electrolytes, amino acids and vitamins) but without any product we were looking for; (e) they only sold in the store, not online; or (f) they were connected with the website that actually sold it, for example, Amazon.

Of the websites initially found, 210 were analyzed in Spanish and 135 in English. Of these, 14 of the websites in Spanish (6.6%) and 26 in English (19.3%) sold the 4 products selected in this study. If we consider the products individually, the percentages of websites in Spanish and English that sold each of them were similar for ginseng (73.8% and 78.5%, respectively) and caffeine (72.4% and 73.3%, respectively), while for cannabidiol (9.0% and 25.9%, respectively) and curcumin (71.0% and 84.0%, respectively), they were more available on the English websites.

Of the websites reviewed in Spanish, 66.7% had their headquarters/location in Spain, 5.1% in Andorra, and 2.6% in the Netherlands; the rest were American (10.3% in Brazil; 5.1% in Mexico and Argentina; 2.6% in the USA and Uruguay). As for those found in English, 53.8% were from the USA, 23.1% from the UK, 7.7% from India and the rest from various countries such as Singapore, New Zealand, Belgium or Spain.

With respect the type of website, we found that the percentages were similar in the two searches for supermarkets and parapharmacies. In the search in Spanish, a greater number of websites offering these products were located in websites that sold nutritional supplements and sports, pharmacy or herbalist websites, whereas in English, there were more websites that were laboratories or that sold all kinds of products.

Regarding the information contained, the highest percentage corresponded to the administration protocol, both in Spanish and English (54.8% and 61.5%, respectively). It should be noted that on many of them, no reference was made to the length of treatment. In the search in Spanish, we could verify that the websites referred mainly to information on ergogenic aids (31.0%), manufacturing laboratory (27.6%), adverse reactions of the compounds (11.0%) and the country of production (8.6%). The search in English provided different results in terms of the frequency of items: manufacturing laboratory (43.7%), country of production (18.5%), information on ergogenic aids (13.3%) and potential adverse reactions (5.9%).

Very few websites refer to the specific type of sport in which these substances would be indicated (11 websites in Spanish and 7 in English), nor is reference made to the results obtained with the product: We only found this information in 2 of 38 (1.4% in Spanish and 0.7% in English) studies, and even those did not provide scientific evidence of their results. Some of the websites indicated that the supplements promoted recovery after exercise (nine websites in Spanish and three in English) and prevented/avoided/buffered against muscle damage caused by exercise (four websites in Spanish and one in English), providing information on improvements in the short long terms but again without scientific evidence.

Information on each of the products individually is included in Table 2. We were able to verify that in the search in Spanish, the largest number of websites that offered cannabidiol and curcumin were those selling nutritional supplements with a section focused on sports or sports nutrition, ginseng in pharmacies and caffeine in sports websites. In those websites in English, it was observed that the largest offer for ginseng, caffeine and curcumin was in supermarkets and for cannabidiol was in pharmacies.

On Spanish websites, the product with the most units available was caffeine (5699 units), followed by ginseng (4896 units) and curcumin (2769 units), with cannabidiol in last place (95 units). On the English ones, the highest supply was for ginseng (3694 units), followed by caffeine (3226 units), curcumin (2866 units) and lastly cannabidiol (739 units). The difference in the number of units available for cannabidiol in Spanish (95 units) and in English (739 units) is also noteworthy.

In relation to the composition of the products containing ginseng, in the search in Spanish, 48.5% of them offered it alone; in 45.0% of them, it was associated with other components, and in 6.5% of them, the composition could not be known. In the English search, it was observed that the majority (83.0%) of the products contained only ginseng. The associations were mainly with jelly, taurine, carnitine, guarana, caffeine, tyrosine, choline, green tea or mate. In the case of caffeine, 51.3% and 76.5% of the products, respectively, contained only this compound in the searches in Spanish and English. The caffeine associations contained mainly carbohydrates, amino acids, B vitamins, vitamin C and minerals. Curcumin was combined with other substances in most cases (79.0% and 81.4% of cases in searches in Spanish and English, respectively). In contrast, on both the English and Spanish websites, CBD was never found associated with other substances.

The dosages of the products varied widely (see Table 2). However, the most frequently offered form for all products in both searches was capsules, except for cannabidiol in the English search, which was oily solutions (42.8%) followed by capsules (33.7%). For the rest of the products, 31.6% of those containing cannabidiol in Spanish were offered as capsules; 50.1% and 45.4% for ginseng in Spanish and English, respectively; 35.2% and 32.4% for caffeine in Spanish and English, respectively, and 62.4% and 69.0% for curcumin in Spanish and English, respectively. The second most offered pharmaceutical forms were compressed tablets for ginseng (17.2% and 9.1% of the websites in Spanish and English, respectively) and curcumin (16.1% and 8.7% of the websites in Spanish and English, respectively); oral drops for cannabidiol in Spanish (26.3%) and powder for caffeine (23.0% and 31.1% of websites in Spanish and English, respectively).

## 4. Discussion

The study aims to evaluate the availability of four supplements in sports use through the internet and analyze the characteristics of the websites that market them. This study assessed the use of caffeine, ginseng, turmeric and cannabidiol as ergogenic aids.

Nutritional supplements are used as ergogenic aids with the purpose of achieving better performance, modifying body composition, promoting muscle development, losing weight or body fat or delaying the onset of fatigue [2,7]. Their consumption is not only a common practice in the general population but also among athletes, who seek through their intake to achieve nutritional or sports objectives, assuming no risk in their intake because they are natural products [49]. As they are products derived from herbs, they tend to be accepted by athletes due to their natural origin, without considering the interactions that many of these active ingredients have with medicines or the contamination or adverse effects if consumed in an inadequate dose [49].

In one study, approximately 64% of the population over 6 years performed some physical activity, and in some cases, they used supplements to reach their sports objectives [8]. Thomas et al. [61] interpreted the increase in supplement consumption as a result of the greater interest among recreational athletes and the general population in leading healthier lifestyles.

The consumption of dietary supplements is more frequent in elite athletes, followed in a smaller proportion by active people and people in general [58]. The preferred supplements among athletes are proteins and multivitamins [62]. Consumption is higher in men and increases with age [59]. As some authors point out, the sports nutrition market involves a variety of products with various presentations, such as powder, capsules, gels, syrups, nutritional bars and energy drinks among others [63]. In our study we have been able to verify the great variety of pharmaceutical forms in which these products are available.

The results show that there are many websites (350) where one or more of the products analyzed (caffeine, turmeric, ginseng and cannabidiol) can be purchased. Although the numerous vendors assessed favor easy and quick purchase of the analyzed products and, consequently their consumption, most of these websites fail to give complete and quality health information on these products. Regarding the origin of the information on the basis of which athletes decide to take a supplement, it is known that it does not come from studies of scientific relevance [64]. The latter explains the misuse in doses and moment of intake for all the supplements, a fact that adds to the lack of professional supervision when advising their incorporation into the daily intake [65]. Finally, other risks should be considered when these products are acquired without the corresponding certification, as contamination by doping agents or harmful substances may be present, or the active ingredient simply lacking [66].

In recent years, the internet has become the market of choice for the purchase of these products due to the advantages it offers [62]. Specifically, the websites in Spanish corresponds to those that sell supplements or sports websites and those in English belong to pharmacies [66,67], parapharmacies or laboratories with online sales. In our study, the top three website types selling these herbal supplements were those focused on nutritional supplements with a sport nutrition section (18.6%), supermarkets (15.7%) and sport websites (14.8%). Thus, approximately one third of the websites evaluated were sport-related, which gives an idea of the current importance of herbal supplements in this field.

One of the most important points of the study is understanding the information available, especially for athletes, to select a supplement and know how to consume it safely.

The websites analyzed provided the administration protocols without including the complete treatment procedures. This particular point may be relevant in the supplements studied because in the case of cannabidiol, research in humans is still needed to establish safe consumption protocols [68]. In a study [69] conducted on rugby players, it was concluded that 25% of them had consumed cannabidiol, 40% to relieve pain or improve sleep. What was relevant was that the athletes turned to internet websites (73%) to find out about the use of the supplement, and only 4% consulted with a professional. It is essential to conduct high-quality research that assesses effects of cannabidiol in sport [68,70], including dose–response studies in which the time of consumption is established according to the sport or the appropriate concentration to achieve a positive effect on performance throughout the sports season. It should also be noted that cannabidiol supplements are less present on the websites analyzed in comparison with the other three products (15.7%), probably because its use among athletes is more recent. Cannabidiol was removed from the list of prohibited substances by the World Anti-Doping Agency (WADA) in or out of competition in 2018, allowing its use by athletes.

Undoubtedly, caffeine is among the most studied supplements in relation to consumption and sports [71]. The multiple benefits of this product in sports practice are well-known, such as improvement in performance and wakefulness [42,71,72]. It is available in capsules, energy drinks, energy gels, aerosols, energy bars, chocolate, candies, chewing gum and beverage form [73]. In our study, whereas capsules and tablets were the most offered pharmaceutical forms for the other three supplements (cannabidiol, ginseng and turmeric), powder was the second most found for caffeine as this active ingredient may be included in protein powders. The different presentations provide a wide range of doses, from 1 mg to more than 300 mg. [74]. Possibly because it has been widely studied, this supplement is readily available on the multiple websites researched.

Ginseng and turmeric are the next in availability. In the first case, it is one of the most widely used medicinal herbs in Asia, with widely demonstrated health benefits, particularly due to its flavonoid content that favors blood circulation [12,75,76]. In the sports field, its relevance is attributed to the activation of relaxing factors in the endothelium [11,76,77].

Finally, turmeric has been studied as a treatment in diseases where pharmacological therapy was not possible [78]. Its consumption is associated with an improvement in aerobic capacity and gastrointestinal functions; it also delays fatigue and has antioxidant properties [24,79,80].

The supplements analyzed are derived from a medicinal herb containing an active ingredient that confers benefits not only for health but also in sports practice [1,2]. From the results obtained, it has been observed that most athletes may acquire the products and information about them from websites. They provide poor information regarding the dosage, which comes from little scientific evidence. It should be borne in mind that although they are considered of natural origin, they are not exempt from containing doping agents or harmful substances. On the other hand, it is necessary to consider that there is still a need to develop high-quality scientific studies, mainly to define consumption protocols. These should include the benefits depending on the practice of sports but also on the objectives to be achieved from the metabolic or nutritional point of view.

Of concern is the lack of information on the supplement safety, more specifically on their potential adverse events, contraindications or interactions, which were infrequently described (5.9–11% depending on the language of the website). This may give a feeling to consumers of being safer than they really are regarding their safety and risks. The advantages of purchasing through the internet include the ready access to these products, the variety of supplements offered, their low cost, and consumer privacy. All these factors favor self-prescription [81,82] or consumption after recommendation by other athletes. Thus, health and sport professionals should provide appropriate scientific information on the benefits and risks of using herbal supplements.

### Limitations and Future Perspectives

This study is not without limitations. The websites described were located at a point in time, so these data should be updated on a regular basis as online offer is constantly changing. Additionally, some websites can disappear and new ones may appear. Moreover, websites in languages other than English and Spanish were not included, and they may also offer these substances. This study serves as a backbone for future research related to the proposed topic.

## 5. Practical Application

From the results obtained, it is recommended that the websites selling these supplements provide information on quality and quantity so as not to compromise the health of those who acquire them, as well as ensure the effectiveness that their consumption entails. Populations should also be educated on good consumption practices, as in many cases these products do not have the appropriate certification, which not only means the absence or deficit of the active ingredients but possible contamination with prohibited and/or harmful substances. Finally, the relevance of discerning which brands and/or websites that provide science-based information should also be emphasized.

## 6. Conclusions

The study highlights the easy access to these supplements through the internet, which may potentially increase the consumption of medicinal herbs in sport. The results show the lack of information available to ensure correct dosages of these products. In this sense, the websites accessed should add basic relevant data such as dosage, time of consumption, length of treatment, as well as the outcomes to be achieved with a certain product.

Finally, it should be considered the lack of scientific evidence to support the use of any of these supplements in relation to sport. Studies carried out with medicinal herbs are scarce and developed in very different conditions regarding the species studied or the amount consumed, and many of them do not identify the active ingredient.

## Figures and Tables

**Table 1:** Characteristics of the websites reviewed.

	Search in Spanish	Search in English	Total
Number of websites reviewed	237	173	410
Number of websites discarded	27	38	65
Number of websites included in the study	210	135	345
Number of websites selling the 4 products	14	26	40
Number of websites selling cannabidiol	19	35	54
Number of websites selling ginseng	155	106	261
Number of websites selling caffeine	152	99	251
Number of websites selling curcumin	149	114	263
Number of websites per type:			
They sell nutritional supplements (They have a sports or sports nutrition section)	45	19	64
Sports	31	20	51
Supermarket	28	26	54
Pharmacy	37	11	48
Parapharmacy	20	19	39
Laboratory	12	17	29
Herbalist	28	7	35
Websites that sell products of all kinds	9	16	25
Number of websites reporting on manufacturing laboratory	58	59	117
Number of websites with information on country of production	18	25	43
Number of websites reporting on ergogenic aids, nutritional supplements for sports	66	18	84
Number of websites reporting on the protocol administration	115	83	198
Number of websites reporting adverse reactions	23	8	31

**Table 2:** Characteristics of the offer for each compound in the websites reviewed.

	Cannabidiol			Ginseng			Caffeine			Curcumin		
	Spanish	English	Total	Spanish	English	Total	Spanish	English	Total	Spanish	English	Total
Number of websites per type:												
They sell nutritional supplements (they have a sports or sports nutrition section)	5	7	12	18	15	33	22	18	40	39	15	54
Sports	1	2	3	26	14	40	30	16	46	27	13	40
Supermarket	0	4	4	22	20	42	26	20	46	24	25	49
Pharmacy	3	11	14	31	10	41	28	8	36	32	9	41
Parapharmacy	4	1	5	18	16	34	17	16	33	7	18	25
Laboratory	0	1	1	8	13	21	6	10	16	4	15	19
Herbalist	3	4	7	24	4	28	16	5	21	11	5	16
Websites that sell products of all kinds	3	5	8	8	14	22	7	6	13	5	14	19
Number of units sold	95	739	834	4896	3694	8590	5699	3226	8925	2769	2866	5635
Pharmaceutical forms												
Capsules	30	249	279	2455	1677	4132	2006	1045	3051	1728	1977	3705
Compressed tablets	17	3	20	843	336	1179	686	237	923	447	250	697
Molding tablets	0	1	1	261	279	540	206	172	378	94	144	238
Vials	0	0	0	274	124	398	34	6	40	26	0	26
Blisters	0	0	0	207	134	341	64	1	65	19	17	36
Sachets	0	0	0	48	11	59	115	58	173	46	9	55
Solution	0	10	10	164	278	442	50	18	68	156	14	170
Bars	0	1	1	66	3	69	200	104	304	13	17	30
Drink	14	6	20	79	163	242	150	177	327	0	10	10
Gel drink	0	1	1	46	75	121	612	286	898	1	61	62
Gummies	0	66	66	13	23	36	112	67	179	20	81	101
Powder	0	8	8	211	234	445	1309	1002	2311	183	237	420
Root	0	0	0	38	123	161	0	1	1	0	0	0
Cream	4	36	40	16	25	41	0	0	0	0	0	0
Oil/oily solution	3	316	319	0	0	0	0	0	0	3	3	6
Tincture	0	11	11	0	0	0	0	0	0	2	2	4
Infusion	0	0	0	47	55	102	25	15	40	0	4	4
Chewing gum	0	0	0	16	18	34	21	13	34	0	0	0
Seed	0	0	0	9	16	25	0	0	0	0	0	0
Oral drops	25	25	50	40	32	72	18	12	30	8	18	26
Oral spray	2	4	6	0	0	0	0	0	0	0	1	1
Others	0	2	2	63	88	151	91	12	103	23	21	44

## Data Availability

Not applicable.

## Appendix A

**Table A1:** STROBE Statement—Checklist of items that should be included in reports of cross-sectional studies.

	Item No.	Recommendation	Page No.
Title and abstract	1	(*a*) Indicate the study’s design with a commonly used term in the title or the abstract	1
		(*b*) Provide in the abstract an informative and balanced summary of what was done and what was found	1 lines 19–27
**Introduction**			
Background/rationale	2	Explain the scientific background and rationale for the investigation being reported	1–3 lines 32–107
Objectives	3	State specific objectives, including any prespecified hypotheses	3 lines 108–111
**Methods**			
Study design	4	Present key elements of study design early in the paper	3 lines 113–127
Setting	5	Describe the setting, locations, and relevant dates, including periods of recruitment, exposure, follow-up, and data collection	3 lines 129–142
Participants	6	(*a*) Give the eligibility criteria, and the sources and methods of selection of participants	N/A
Variables	7	Clearly define all outcomes, exposures, predictors, potential confounders, and effect modifiers. Give diagnostic criteria, if applicable	N/A
Data sources/measurement	8 *	For each variable of interest, give sources of data and details of methods of assessment (measurement). Describe comparability of assessment methods if there is more than one group	3–4 lines 113–121, 144–184
Bias	9	Describe any efforts to address potential sources of bias	3 lines 119–120
Study size	10	Explain how the study size was arrived at	N/A
Quantitative variables	11	Explain how quantitative variables were handled in the analyses. If applicable, describe which groupings were chosen and why	N/A
Statistical methods	12	(*a*) Describe all statistical methods, including those used to control for confounding	N/A
		(*b*) Describe any methods used to examine subgroups and interactions	N/A
		(*c*) Explain how missing data were addressed	N/A
		(*d*) If applicable, describe analytical methods taking account of sampling strategy	N/A
		(*e*) Describe any sensitivity analyses	N/A
**Results**			
Participants	13 *	(a) Report numbers of individuals at each stage of study—e.g., numbers potentially eligible, examined for eligibility, confirmed eligible, included in the study, completing follow-up, and analysed	4 lines 186–188
		(b) Give reasons for non-participation at each stage	4 lines 188–193
		(c) Consider use of a flow diagram	N/A
Descriptive data	14 *	(a) Give characteristics of study participants (e.g., demographic, clinical, social) and information on exposures and potential confounders	5 lines 197–208
		(b) Indicate number of participants with missing data for each variable of interest	N/A
Outcome data	15 *	Report numbers of outcome events or summary measures	Table 1 and Table 2
Main results	16	(*a*) Give unadjusted estimates and, if applicable, confounder-adjusted estimates and their precision (e.g., 95% confidence interval). Make clear which confounders were adjusted for and why they were included	N/A
		(*b*) Report category boundaries when continuous variables were categorized	N/A
		(*c*) If relevant, consider translating estimates of relative risk into absolute risk for a meaningful time period	N/A
Other analyses	17	Report other analyses done—e.g., analyses of subgroups and interactions, and sensitivity analyses	N/A
**Discussion**			
Key results	18	Summarise key results with reference to study objectives	10–11 lines 292–342
Limitations	19	Discuss limitations of the study, taking into account sources of potential bias or imprecision. Discuss both direction and magnitude of any potential bias	8 lines 343–347
Interpretation	20	Give a cautious overall interpretation of results considering objectives, limitations, multiplicity of analyses, results from similar studies, and other relevant evidence	N/A
Generalisability	21	Discuss the generalisability (external validity) of the study results	N/A
**Other information**			
Funding	22	Give the source of funding and the role of the funders for the present study and, if applicable, for the original study on which the present article is based	The study was not funded

* Give information separately for exposed and unexposed groups. Note: An Explanation and Elaboration article discusses each checklist item and gives methodological background and published examples of transparent reporting. The STROBE checklist is best used in conjunction with this article (freely available on the Web sites of PLoS Medicine at http://www.plosmedicine.org/ (accessed on 23 March 2022), Annals of Internal Medicine at http://www.annals.org/ (accessed on 23 March 2022), and Epidemiology at http://www.epidem.com/ (accessed on 23 March 2022). Information on the STROBE Initiative is available at www.strobe-statement.org (accessed on 5 June 2022).
